# The pH-sensitive translocation of V-ATPase in the small airway of cystic fibrosis pigs

**DOI:** 10.1152/ajplung.00147.2024

**Published:** 2024-09-10

**Authors:** Raul Villacreses, Ian M. Thornell, Xiaopeng Li, Steven E. Mather, Jimena Urbano, David A. Stoltz, Joseph Zabner

**Affiliations:** ^1^Department of Internal Medicine, Roy J. and Lucille A. Carver College of Medicine, University of Iowa, Iowa City, Iowa, United States; ^2^Department of Pediatrics and Human Development, College of Human Medicine, Michigan State University, Grand Rapids, Michigan, United States

**Keywords:** airway surface liquid, cystic fibrosis, pH, small airways, V-ATPase

## Abstract

In contrast to pig large airways, the pH of airway surface liquid (ASL) in pig small airways is regulated by CFTR-mediated HCO^–^_3_ secretion and the vacuolar-type H^+^ ATPase (V-ATPase) proton secretion. We hypothesized that, in cystic fibrosis (CF), the ASL pH of small airways is acidic, and the V-ATPase is internalized. We quantified proton secretion during the addition of an alkaline test solution by measuring changes in a pH-dependent fluorescent dye generated by porcine small airway epithelia in the absence and presence of bafilomycin A1. The pH-dependent translocation of V-ATPase in ex vivo and in vivo preparations was measured using immunolocalization of V-ATPase. We found that bafilomycin-sensitive proton secretion stopped when the ASL pH was less than 7.10. In non-CF pigs and mice, we found that V-ATPase was localized in the apical membrane, and internalized when the lungs were instilled with a pH 6.8 solution. Studies in which we immediately fixed lungs from pigs revealed apical V-ATPase detection in non-CF piglets and less apical detection in CF piglets. Our data suggest that V-ATPase in small airways is internalized when the ASL pH is acidic. The decrease in apical localization of V-ATPase in CF pigs is consistent with an acidic ASL pH.

**NEW & NOTEWORTHY** In this study, we describe that vacuolar-type H^+^ ATPase (V-ATPase) internalizes when the airway surface liquid (ASL) pH in pig small airways is less than 7.10. Furthermore, we found that V-ATPase is not localized to the apical membrane in the small airways of newborn cystic fibrosis pigs.

## Graphical abstract

**Figure:**
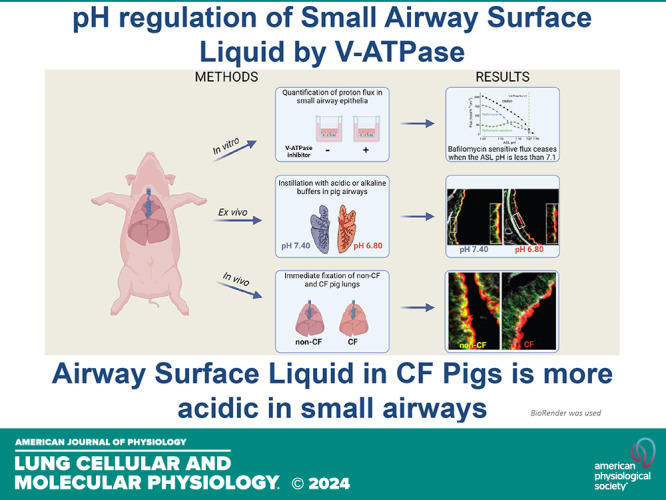

## INTRODUCTION

Cystic fibrosis (CF) is a genetic disease in which cystic fibrosis transmembrane conductance regulator (CFTR) anion channels fail to move Cl^–^ and HCO^–^_3_ through cell membranes. CFTR is expressed throughout the airway epithelium, including small airways. The CF pig recapitulates features of human CF airway disease including dysfunctional mucociliary transport (MCT) (1–3) and deficient bacterial killing (4). These CF hallmarks occur even in the absence of infection and inflammation. Results from in vivo experiments performed on large airways indicate that the lack of HCO^–^_3_ secretion through CFTR acidifies the airway surface liquid (ASL) and in turn impairs host defenses. These in vivo experiments cannot be performed on small airways because of their size.

Human small airways are defined as airways of less than 2 mm internal diameter containing a cuboidal epithelium and lacking cartilage and submucosal glands (5, 6). Consistent with an early role in airway disease progression, autopsy reports for children with cystic fibrosis (CF) show mucus obstruction, bronchiolar wall remodeling, and stenosis within their small airways (7). Although small airways often show pathological changes in the early stages of airway disease, more than 75% of the surface area of the small airways needs to be compromised before eliciting substantial airflow resistance. For this reason, small airways are called the “silent zone” of the lungs (8).

Studies using epithelia cultured from small airways indicate that their ASL is acidic in CF (9). Cultured airway epithelia also provided a mechanism. Although the acidification of large airways arises from the failure of CFTR to neutralize acid secretion by the H^+^-K^+^ pump ATP12A, this pump is absent in small airways (9). Acidification of small airway ASL arises in part from the presence of the V-type H^+^ pump (V-ATPase). However, it remains unknown if small airways are acidic in vivo.

The V-ATPase hydrolyzes ATP to move H^+^ from the cytosol into the ASL. We previously reported that small airway epithelia express the V-ATPase in the plasma membrane and contribute to the acidification of ASL observed in cultured CF small airways (9). Here, we evaluate the pH sensitivity of the V-ATPase activity in small airway epithelia of pigs. Then, we use freshly excised tissue from pigs and mice to test whether the V-ATPase is regulated by pH across distinct species. Finally, V-ATPase localization was used to determine whether the small airways of newborn CF pigs are acidic.

## MATERIALS AND METHODS

### Primary Cell Cultures of Pig Small Airway Epithelia

Animal studies were approved by the University of Iowa Animal Care and Use Committee. Small airway epithelia were dissected and cultured as previously described (9).

### Rate of Acidification

Apical surfaces of cultured airway epithelia were washed with HCO^–^_3_-free assay solution [(in mM) 135 NaCl, 2.5 HEPES free acid, 1.2 CaCl_2_, 1.2 MgCl_2_, and 30 KCl; pH 7.60]. The epithelia were placed in prewarmed Krebs solution [(in mM) 135 NaCl, 5 HEPES free-acid, 2.4 K_2_HPO_4_, 0.6 KH_2_PO_4_, 1.2 CaCl_2_, 1.2 MgCl_2_; pH 7.40], and then 30 µL of assay solution with DMSO or 200 nM bafilomycin A1 was added to the apical surface. After 10 min, the apical solution was aspirated, and the epithelia were moved to a humified chamber containing basolateral Krebs solution for imaging with a Zeiss LSM880 confocal microscope. Then, 30 µL of HCO^–^_3_-free assay solution containing 5 mg·mL^−1^ SNARF-1 70 kDa dextran was added to the apical surface, and pH-dependent fluorescence was monitored by exciting SNARF-1 with a 514 nm laser and collecting emissions at 580 nm and 640 nm as previously described (10). SNARF-1 fluorescence was monitored until a steady-state fluorescence was reached, usually ∼30 min. SNARF-1 fluorescence was converted to pH values using GraphPad Prism software and a modified acid-base titration equation (11):

$$\text{ratio observed }=a+b\left(\frac{10^{\left(\text{pH}-pK_{\text{a}}\right)}}{1+10^{\left(\mathrm{pH}-pK_{a}\right)}}\right)$$

where “ratio observed” is the monitored 580/640 emission and variables *a*, *b*, and pK_a_ are obtained from fitting. Variables pH and pK_a_ have their chemical definitions.

To eliminate noise, experimental plots of pH versus time were smoothened using the LOWESS fitting method. The smoothened data were used to calculate the rate of pH change (dpH/dt) for each pH value throughout the experiment. Proton flux (ϕ) was then calculated for each pH value from the fundamental law of pH regulation (12):

$$\frac{\mathrm{dpH}}{\mathrm{dt}}=\frac{A\times \Phi }{V(\beta )}$$

where dpH/dt is the calculated rate of pH change at an instantaneous pH value, *A* is the epithelial surface area (0.33 cm^2^), V is the apical volume (30 µL), ϕ is the calculated flux (mol^−1^·h^−1^), and β is the pH-dependent buffering capacity of the apical assay solution calculated by first computing the buffering capacity of each buffer at a given pH value (13–16):

$$\beta =[\mathrm{buffer}]\frac{2.3\times K_{a}\times [{\text{H}}^{+}]}{{\left(K_{a}+[{\text{H}}^{+}]\right)}^{2}}$$

where *K*_a_ is the dissociation constant of the buffer. Two buffers are present in our experiments: 2.5 mM HEPES and 14.3 µM SNARF-1 (based on the manufacturer’s estimated stoichiometry of SNARF-dextran conjugation). Therefore, the total pH-dependent buffering capacity was computed by adding β for each buffer at a given pH value. Note that errors in the SNARF-dextran stoichiometry will have a nominal effect on the computed buffering capacity because [HEPES] ≫ [SNARF-1]. *K*_a_ values were computed using pK_a_ values at 37°C; 7.30 for HEPES (17) and 6.90 for SNARF-1.

### Porcine Tissue Preparation and Treatments

After euthanizing the animal, the lungs were removed and the main bronchi were cannulated. For our experiments, the right and left lungs were randomly instilled with either HEPES buffer [(in mM) 135 NaCl, 2.4 K_2_HPO_4_, 0.6 KH_2_PO_4_, 1.2 CaCl_2_, 1.2 MgCl_2_, 10 mM HEPES, and 10 mM glucose, pH 7.40] or PIPES buffer [(in mM) 135 NaCl, 2.4 K_2_HPO_4_, 0.6 KH_2_PO_4_, 1.2 CaCl_2_, 1.2 MgCl_2_, 10 mM PIPES, and 10 mM glucose, pH 6.80] until the lung parenchyma was fully expanded. After instillation, the cannulas were clamped and secured with hemostats. The lungs were sealed in a bag and kept at 37°C for 60 min in a water bath. We then aspirated the buffer solutions and instilled 4% paraformaldehyde (PFA) until the lung parenchyma was fully expanded.

For non-CF and CF porcine baseline experiments, the animal was euthanized, the trachea immediately cannulated, and 4% PFA was instilled until we obtained full expansion of the lung parenchyma.

A total of four different cuts, two central and two peripheral, were incubated in 4% PFA overnight. The tissues were then exposed to a sucrose gradient. Finally, the tissues were embedded in Tissue-Tek O.C.T. Compound (Sakura 4583) for cryosectioning.

### Mouse Experiments and Tissue Preparation

After the animal was euthanized, the trachea was cannulated, the lungs were expanded with either HEPES buffer (pH 7.40) or PIPES (pH 6.80), and the cannula was clamped and secured with nylon sutures. The lungs were kept sealed in a bag for 60 min at 37°C in a water bath and then the tissues were fixed and prepared as previously described.

### Immunohistochemistry

Pig cryosections were permeabilized with 0.25% Triton X, and then blocked with superblock for 30 min followed by 5% normal goat serum in TBS-T for 45 min. Samples were then incubated with mouse anti-*ATP6V0D2* (ABCAM ab194557) at 1:100 dilution and rabbit anti-*MUC1* (ABCAM ab15481) at 1:100 dilution overnight at 4°C. Then, the samples were incubated with the secondary antibodies: goat-anti-mouse-Alexa-Fluor-488 and goat anti-rabbit-Alexa-Fluor-568 (Thermo Fisher) at a 1:1,000 dilution. Finally, the samples were also incubated in Phalloidin conjugated with Alexa 647 (Thermo Fisher) at 1:50 dilution. Mouse cryosections were permeabilized and blocked as previously described, with the exception of using 5% normal donkey serum. Samples were then incubated with rabbit anti-*ATP6V0D2* (Invitrogen PA5-63109) at 1:100 and goat anti-*SCGB3A2* (R&D Systems AF3545) at a 1:100 dilution overnight followed by incubation with secondary antibodies donkey-anti-rabbit-Alexa-Fluor-488 and donkey-anti-goat 568 (Thermo Fisher) at a 1:1,000 dilution. Phalloidin conjugated with Alexa 647 at a 1:50 dilution was also used. All samples were mounted with DAPI (Vectashield, Vector Laboratories) for nuclear counterstaining. Fluorescence was visualized using an Olympus Fluoview FV3000 confocal microscope, using a UPLSAPO ×20 oil lens.

To quantify the apical fluorescence intensity of V-ATPase, we created a region of interest (ROI) using the apical fluorescence of Mucin 1 (MUC1) with FIJI (ImageJ). We then determined the corrected total cell fluorescence (CTCF) in arbitrary units for MUC1 and the V0d2 subunit of V-ATPase using the following formula (18): CTCF = integrated density − (area of ROI × mean fluorescence of background readings).

For the quantification of apical V-ATPase in murine samples, we first created an arbitrary apical ROI using FIJI. We then measured the CTCF for Secretoglobin family 3A member 2 (SCGB3A2) and V0d2 as previously described.

### Statistics

Statistical analysis to compare the difference between the groups from all in vivo and ex vivo experiments was performed by using the nonparametric Mann–Whitney *U* test. Bars in figures represent standard error of the mean.

## RESULTS

### V-ATPase Activity Is pH-Dependent in Porcine Small Airway Epithelia

**Figure 1.**
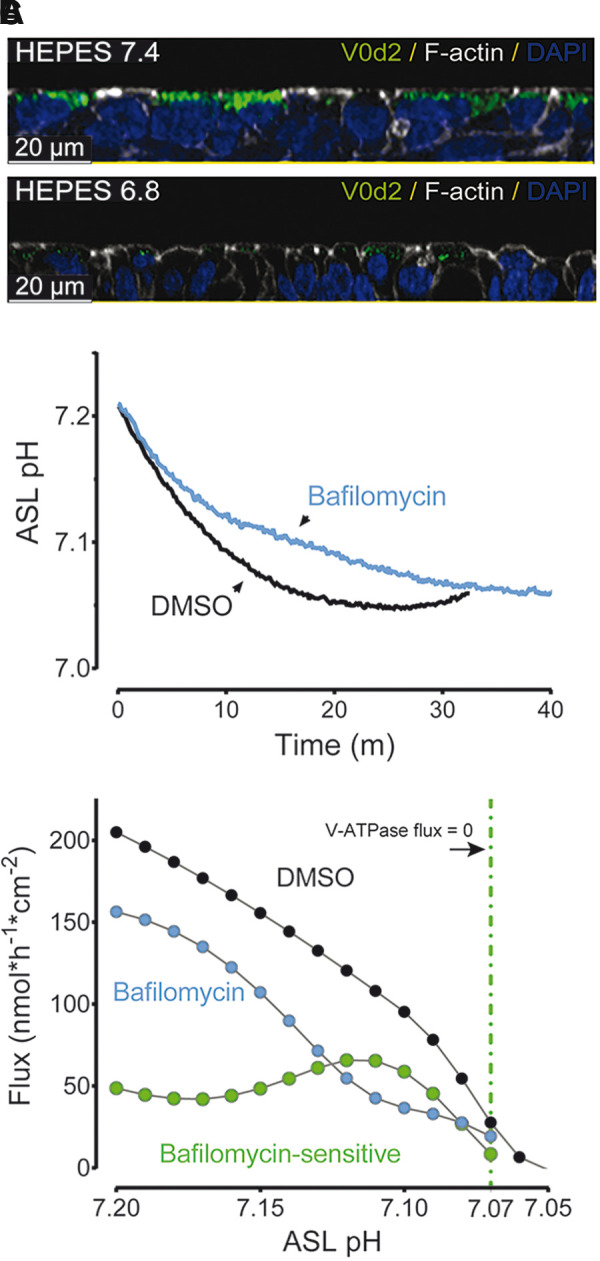
Apical localization of vacuolar-type H^+^ ATPase (V-ATPase) decreases when the airway surface liquid (ASL) pH is more acidic in porcine small airway epithelia (*A*). *B*: the rate of acidification is delayed in epithelia treated with bafilomycin A1. *C*: bafilomycin-sensitive H^+^ flux (V-ATPase) ceases when the airway surface liquid pH is less than 7.07.

The V-ATPase internalizes between a pH of 7.40 and 6.80 in several epithelia. Using pig small airway epithelia cultured at the air-liquid interface, we evaluated the level of apical V-ATPase expression where the ASL pH was clamped to either 7.40 or 6.80 (Supplemental Fig. S1). At pH 7.40, V-ATPase could be detected in occasional cells (Fig. 1*A*). At an ASL pH of 6.80, V-ATPase is no longer localized at the apical membrane. The V0d2 subunit of the V-ATPase enables its apical localization and we previously demonstrated, with immunocytochemistry, that this subunit is expressed by secretory cells of the small airways of pigs (9).

To investigate the ASL pH threshold for internalization, we measured the rate of acidification of an apically applied pH 7.40 solution in the absence of CO_2_/HCO^–^_3_. Small airway epithelia acidified the solution over 20 min and the addition of the V-ATPase inhibitor bafilomycin slowed the acidification rate (Fig. 1*B*). Using the obtained acidification rates (dpH/dt), apical volume, and the buffering capacity of the solution, we calculated the bafilomycin-sensitive fluxes (V-ATPase) at different apical pH values (Fig. 1*C*). When the pH of the test solution was greater than 7.10, the H^+^ fluxes were ∼50 nmol·h^−1^·cm^−2^, a finding that is consistent with active pumping of H^+^ into the ASL rather than ion gradients. As pH decreased beyond 7.10, the bafilomycin-sensitive flux approached zero indicating that V-ATPase activity ceased below this pH value. The flux data and the immunofluorescent data suggest that the V-ATPase is internalized near a pH of 7.10.

### Acidification Decreases Apical V-ATPase in Murine Large Airways

**Figure 2.**
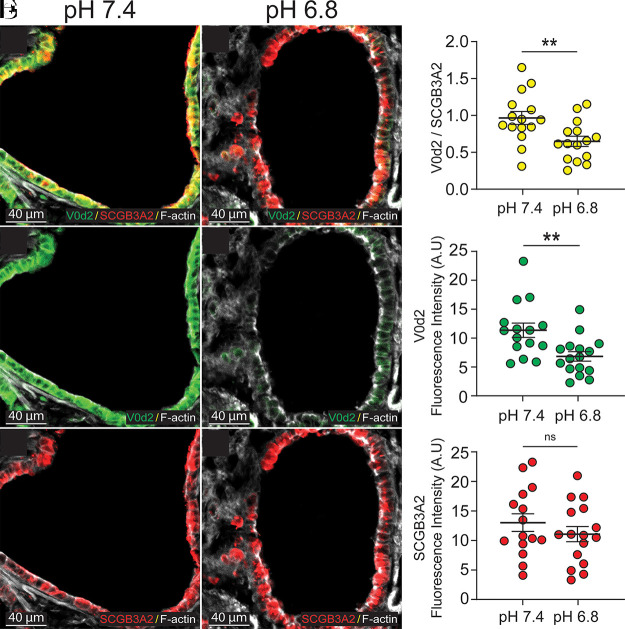
Images of mouse airways treated with buffers set to either pH 7.40 or 6.80. Data were obtained from multiple regions of interest (ROI) per airway from four different mice. *A*, *D*, and *G*: colocalization (yellow) of V0d2 and SCGB3A2. *G* shows a significantly higher average ratio at pH 7.4. **P = 0.002. *B*, *E*, and *H*: immunolocalization for V0d2 (green). *H* shows fluorescence is significantly higher when the luminal pH is alkaline. **P = 0.008. *C*, *F*, and *I*: immunolocalization for SCGB3A2 (red). *I* shows that SCGB3A2 is pH insensitive.

V0d2, the subunit of the V-ATPase associated with H^+^ secretion across apical membranes, is also found in the large airways of mice. Mice large airways lack submucosal glands (a few are present near the larynx) and measure between 200 and 400 µm (19). To test whether pH regulation of the V-type H^+^ pump occurred across species where cartilaginous airways express the V-type H^+^ pump, we instilled murine lungs with either a pH 7.40 or 6.80 solution for 60 min and then detected V0d2 by immunofluorescence. V0d2 signal was significantly more intense at pH 7.40 than at pH 6.80 indicating pH-dependent internalization of V-type H^+^ pumps (Fig. 2, *A*–*C*). In contrast, the club cell marker SCGB3A2 was pH-insensitive (Fig. 2, *D*–*F*). These data indicate that pH-dependent detection of V-type H^+^ pumps is conserved in cultured porcine small airway epithelia and ex vivo murine large airways.

### Internalization of V-ATPase in Porcine CF Small Airway Epithelia In Vivo Reveals Acidic Small Airways in CF

**Figure 3.**
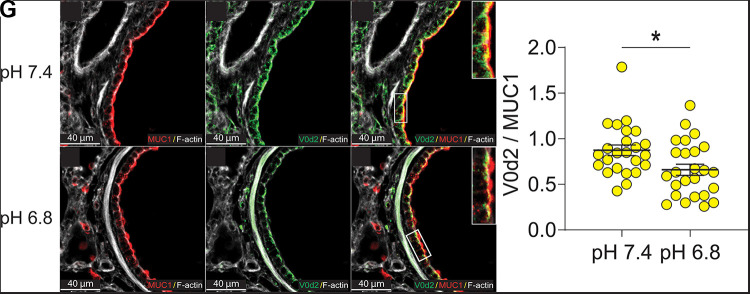
Images of non-CF pig small airways treated with buffers set to either pH 7.40 or 6.80. *A* and *D*: immunofluorescence for MUC1 (red) does not change at different luminal pH. *B* and *E*: apical localization of V0d2 (green) decreases when the luminal pH is acidic. *C* and *F*: colocalization of V0d2 and MUC1. *G*: average ratio of multiple regions of interest (ROIs) per airway, from four different pigs. *P = 0.02. CF, cystic fibrosis.

Porcine V-type H^+^ pumps of cultured airway epithelia internalize near a pH of 7.10. Therefore, V-type H^+^ pump localization can indicate whether the ASL pH is above or below 7.10. Using this knowledge, we tested whether CF small airways are acidic in vivo. First, we evaluated whether the V-type H^+^ pump internalized ex vivo. In a single lung, we instilled one lobe with a pH 7.40 solution and another with a pH 6.80 solution. After 60 min, the lungs were fixed and stained for the V0d2 subunit (Fig. 3, *B* and *E*) and the cytoplasmic tail of mucin 1 to label the apical membrane (Fig. 3, *A* and *D*). As in vitro, we observed robust V0d2 localization in the apical membrane at pH 7.40 and less at pH 6.80 (Fig. 3*G*).

**Figure 4.**
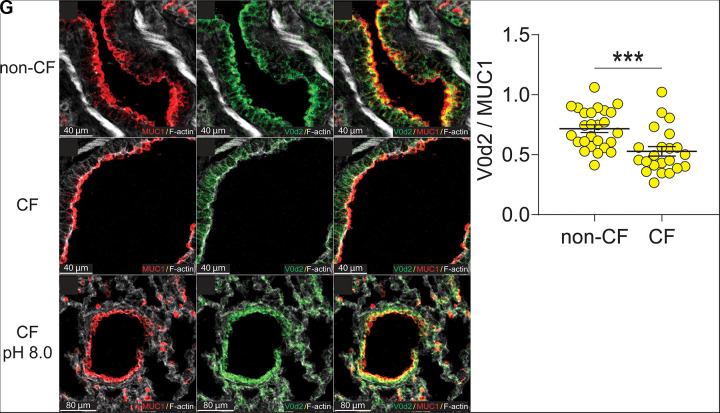
Images of non-CF and CF pig small airways. *A* and *D*: immunofluorescence for MUC1 (red) is not different between non-CF and CF. *B* and *E*: apical localization of V0d2 (green) is significantly lower in CF. *C* and *F*: colocalization of V0d2 and MUC1 in non-CF and CF small airways. *G*: average ratio between V0d2 and MUC1 of multiple regions of interest (ROIs) per airway, from four different pigs. ***P = 0.0002. *H–J*: representative images of co-localization of V0d2 and MUC1 in CF small airways when the luminal pH is alkaline (*n* = 2 pigs). CF, cystic fibrosis.

These data indicate that the pH sensitivity of V-type H^+^ pumps is intact in excised pig tissue. We then evaluated the expression of V0d2 in vivo. Non-CF and CF newborn pigs were euthanized, and the lungs were immediately fixed. For non-CF pigs, we detected a robust V0d2 signal that co-localized with the apical membrane marker mucin-1 (Fig. 4; Supplemental Figs. S2–S4). These data indicate the newborn pig small airways have a pH >7.10. In contrast, CF pigs had a sparse punctate V0d2 signal that rarely co-localized with MUC1. We instilled CF lobes with a pH 8 HEPES solution for 60 min and found robust V0d2 localization in the apical membrane (Fig. 4, *H*–*J*). These data indicate that the small airways of CF pigs are acidic relative to non-CF pigs in vivo.

## SUMMARY AND DISCUSSION

Our current study shows that, as in the clear cells of the epididymis and intercalated cells of the kidney (20), the apical localization of V-ATPase is driven by the ASL pH in mouse airways and pig small airways. We measured the acidification rate of pig small airway epithelia and found that the bafilomycin-sensitive proton flux, which represents V-ATPase activity, halts when the ASL pH is less than ∼7.10. Based on these results, we speculate that pig small airways can sense a low pH, which then triggers translocation of the apical V-ATPase. Furthermore, we found infrequent in vivo apical localization of V-ATPase in CF pigs compared with non-CF pigs indicating that the pH of newborn CF pig small airways is more acidic than in non-CF. When cell membrane proteins are internalized, they no longer remain concentrated at the cell membrane, which affects their visibility in immunocytochemistry. However, we cannot exclude the possibility that low apical pH leads to reduced expression or increased degradation of the V-ATPase.

Initial studies by Fabricant (21) reported that the pH of nasal secretions ranged from 5.50 to 6.50. However, another study by McShane et al. (22) found a broad pH range from pH 5.50 to 8.0 in CF and 5.50 to 7.40 in non-CF subjects in different regions of the proximal airway tree. Finally, Abou Alaiwa et al. (23) also investigated the difference in ASL pH between neonates with and without CF. This study again showed a wide ASL pH range from as low as 4.50 to as high as 7.90. In humans, the arterial blood pH is constant; on average, it is 7.40 and has only small fluctuations between and within individuals. The discrepancy between the fluctuations of the ASL pH and in the blood prompts us to ask more questions like, how is the ASL pH of the airways regulated? and why are these fluctuations needed?

It is assumed that the major regulators of ASL pH in large airways are CO_2_, CFTR-mediated HCO^–^_3_ secretion, and H^+^ secretion by ATP12A. Large airways are exposed to rapid fluctuations of CO_2_ during tidal breathing, from 0.04% during inspiration to 5% during exhalation. The fluctuation of ASL pH has been attributed to the CO_2_ variation due to breathing. However, Fabricant (24) showed that the nasal pH of individual subjects remained constant when it was measured minute by minute, suggesting that CO_2_ plays a less significant role in the homeostasis of ASL pH in large airways. There are conflicting data regarding the role of exhaled CO_2_ in the regulation of ASL pH. We found that lack of soluble carbonic anhydrase in the ASL of pig large airway epithelial cultures results in blunted pH fluctuations at different CO_2_ concentrations (10). However, Dusik et al. (25) showed large ASL pH oscillations (7.50–9.0) during tidal breathing in humans.

Antimicrobial peptide activity represents one example of why these pH fluctuations might be needed. Antimicrobial peptides are ubiquitous molecules that possess a broad antimicrobial spectrum and, interestingly, they share a common feature with antibiotics. They both work better at different pH values. For example, the bactericidal activity of human β-defensin 3 (hBD-3) and the cathelicidin LL-37 is much more robust at higher pH values (26), and this pH-dependent antimicrobial activity is also seen in aminoglycosides, β-lactams, and fluoroquinolones (27).

In small airways, the homeostasis of ASL pH is different than in large airways. Small airways are exposed to a constant concentration of CO_2_ given their proximity to the alveolar space. The control of the ASL pH is largely driven by HCO^–^_3_ secretion through CFTR, but, interestingly, small airways do not express ATP12A. We previously found, in vitro, that secretory cells of pig small airways express the V0d2 isoform (ATP6V0d2) of the V-ATPase and found that its localization to the apical membrane is dependent on the ASL pH. Interestingly, ionocytes also express ATP6V0d2 (28), although its regulation has not been studied. We speculate that expressing ATP6V0d2 allows the V-ATPase, a ubiquitous proton pump, to translocate to the apical membrane and that this translocation is also regulated by similar mechanisms seen in the kidney and epididymis.

Currently, the sensor of ASL pH in the airways is unknown. Potential candidates include intrinsic pH-sensing mechanisms of the V-ATPase and the soluble adenylyl cyclase (sAC). The intrinsic pH-sensing capacity of the V-ATPase has been shown to recognize a low pH in organelles (29).

In clear cells of the epididymis (20), it is proposed that the sAC serves as an indirect sensor of pH as its activity is regulated by luminal HCO^–^_3_ that subsequently gets transported to the cytosol. Activation of the sAC leads to increased levels of cAMP, which subsequently activates the cAMP-dependent protein kinase (PKA) which then promotes translocation of the V-ATPase to the apical membrane by mechanisms that have not been clearly described. This is an important function in the epididymis as an acidic pH is necessary for the maturation and function of spermatozoids.

In other tissues, elevating intracellular cAMP blocks the internalization of the V-ATPase during acidification (30). This feature of the V-ATPase has implications for small airways. For example, β-adrenergic long-acting bronchodilators increase the levels of cAMP in the airways, which would then lead to the activation of PKA and the translocation of V-ATPase to the apical membrane. In CF, β-adrenergic long-acting bronchodilators could further decrease the ASL pH. Interestingly, a male oral contraceptive, TDI-11861, targets V-ATPase in the male reproductive tract by blocking the cAMP-generating enzyme soluble adenyl cyclase (sAC) (31). Airways also express sAC (32). TDI-11861 could lead to an increase in ASL pH by preventing the translocation of V-ATPase. The effect of alkaline pH in small airways is unclear, but the use of V-ATPase to offset HCO^–^_3_ secretion suggests that small airways may have an optimal physiological pH.

## DATA AVAILABILITY

Data will be made available upon reasonable request.

## SUPPLEMENTAL MATERIAL

10.6084/m9.figshare.26322160 Supplemental Figs. S1–S4: https://doi.org/10.6084/m9.figshare.26322160.

## GRANTS

This work was supported by National Center for Advancing Translational Sciences (K12TR004382 to R.V.) and National Heart, Lung, and Blood Institute (HL153165-01A1 to X.L. and J.Z.; HL091842 to R.V., I.M.T., X.L., S.E.M., J.U., D.A.S., and J.Z.).

## DISCLOSURES

The University of Iowa Research Foundation has licensed intellectual property related to gene-modified pigs to Exemplar Genetics. Royalties from that license are shared with D. A. Stoltz. D. A. Stoltz has no other financial ties to Exemplar Genetics. None of the other authors has any conflicts of interest, financial or otherwise, to disclose.

## AUTHOR CONTRIBUTIONS

R.V., I.M.T., X.L., and J.Z. conceived and designed research; R.V., I.M.T., S.E.M., and J.U. performed experiments; R.V., I.M.T., S.E.M., and J.Z. analyzed data; R.V., I.M.T., and J.Z. interpreted results of experiments; R.V., I.M.T., J.U., and J.Z. prepared figures; R.V. and J.Z. drafted manuscript; R.V., I.M.T., X.L., D.A.S., and J.Z. edited and revised manuscript; R.V. and J.Z. approved final version of manuscript.
